# Feline calicivirus p32, p39 and p30 proteins localize to the endoplasmic reticulum to initiate replication complex formation

**DOI:** 10.1099/vir.0.016279-0

**Published:** 2010-03

**Authors:** Dalan Bailey, William J. Kaiser, Mike Hollinshead, Katy Moffat, Yasmin Chaudhry, Thomas Wileman, Stanislav V. Sosnovtsev, Ian G. Goodfellow

**Affiliations:** 1 Department of Virology, Imperial College London, Norfolk Place, London W2 1PG, UK; 2 School of Biological Sciences, University of Reading, Whiteknights, Reading RG6 6AJ, UK; 3 Institute for Animal Health, Pirbright, Ash Road, Guildford GU24 0NF, UK; 4 School of Medicine, University of East Anglia, Norwich NR4 7TJ, UK; 5 Laboratory of Infectious Diseases, National Institute of Allergy and Infectious Diseases, National Institutes of Health, Bethesda, Maryland, USA

## Abstract

In common with other positive-strand RNA viruses, replication of feline calicivirus (FCV) results in rearrangement of intracellular membranes and production of numerous membrane-bound vesicular structures on which viral genome replication is thought to occur. In this study, bioinformatics approaches have identified three of the FCV non-structural proteins, namely p32, p39 and p30, as potential transmembrane proteins. These proteins were able to target enhanced cyan fluorescent protein to membrane fractions where they behaved as integral membrane proteins. Immunofluorescence microscopy of these proteins expressed in cells showed co-localization with endoplasmic reticulum (ER) markers. Further electron microscopy analysis of cells co-expressing FCV p39 or p30 with a horseradish peroxidase protein containing the KDEL ER retention motif demonstrated gross morphological changes to the ER. Similar reorganization patterns, especially for those produced by p30, were observed in naturally infected Crandel–Rees feline kidney cells. Together, the data demonstrate that the p32, p39 and p30 proteins of FCV locate to the ER and lead to reorganization of ER membranes. This suggests that they may play a role in the generation of FCV replication complexes and that the endoplasmic reticulum may represent the potential source of the membrane vesicles induced during FCV infection.

## INTRODUCTION

Members of the family *Caliciviridae* of positive-stranded RNA viruses are important pathogens of both man and animals. Feline calicivirus (FCV) infection generally results in an acute oral and upper respiratory tract disease in all feline species (Gaskell *et al.*, 2004); however, recent isolates have been shown to cause virulent systemic disease (Hurley & Sykes, 2003). Although vaccines to FCV exist, their efficacy against recent field isolates appears to be reduced and interestingly, outbreaks of virulent systemic FCV infection have been reported in vaccinated cats (Hurley & Sykes, 2003). Hence, studies into the development of new vaccines and mechanisms to control virus infection are warranted.

It is generally accepted that the majority of RNA synthesis during the replication of positive-stranded RNA viruses occurs on membrane vesicles formed in the cytoplasm of infected cells (Cottam *et al.*, 2009; Miller & Krijnse-Locker, 2008; Netherton *et al.*, 2007). Recent work using immunogold electron microscopy and immunofluorescence microscopy has revealed that the origin and composition of these vesicles may differ markedly between viruses, and even within virus families. For example, within the family *Picornaviridae*, poliovirus replication has been associated with endoplasmic reticulum (ER)-derived COPII-containing vesicles (Rust *et al.*, 2001) or membranes generated during autophagy (Jackson *et al.*, 2005; Kirkegaard *et al.*, 2004; Suhy *et al.*, 2000), while work with foot-and-mouth disease virus (FMDV) demonstrates that replication occurs on juxtanuclear membranes close to the Golgi that lack ER markers (Knox *et al.*, 2005). Parallel work suggests that proteins responsible for regulating the formation of vesicles in the early secretory pathway are involved in the formation and/or function of replication sites. Brefeldin A (BFA; a fungal metabolite) inhibits the Arf1 GTP exchange factors GBF1 and BIG1/2 that regulate the assembly of COPI coats on vesicles that traffic from the Golgi to the ER. Sensitivity to this drug varies between positive-stranded RNA viruses; enterovirus replication is inhibited by BFA but other picornaviruses are not (Cottam *et al.*, 2009; Gazina *et al.*, 2002). Interestingly, the two well-characterized caliciviruses FCV and murine norovirus (MNV) have also been shown to be insensitive to this drug (Green *et al.*, 2002; Hyde *et al.*, 2009).

Caliciviruses induce membrane-bound replication complexes similar to other positive-stranded RNA viruses (Love & Sabine, 1975; Studdert & O'Shea, 1975), but little is known about the origin, composition and mechanism of formation of the membranes that support the replication complexes. MNV infection of a macrophage cell line produces single and double membraned vesicles (Wobus *et al.*, 2004) that have recently been shown to originate in the perinuclear region and co-localize with components of the early and late secretory pathway (Hyde *et al.*, 2009). Studies with human noroviruses suggest vesicles may result from disassembly of the Golgi apparatus following binding of viral proteins to Vamp-associated-protein A (VAP-A) (Ettayebi & Hardy, 2003; Fernandez-Vega *et al.*, 2004).

**Fig. 1. f1:**
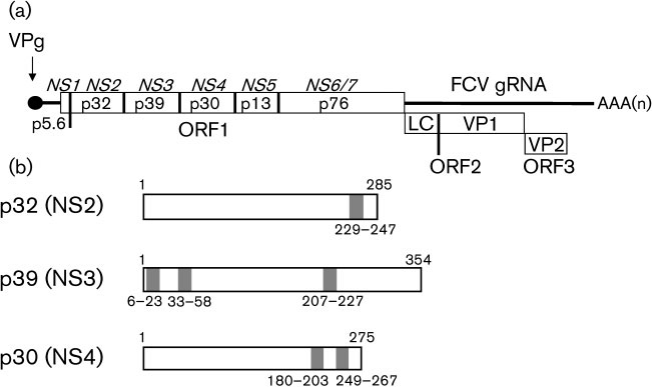
Diagrammatic representation of the FCV genome and the p32, p39 and p30 proteins. (a) Proteolytic cleavage map of FCV as demonstrated by Sosnovtsev *et al.* (2002). The NS1–7 nomenclature, as recently adopted for MNV (detailed by Sosnovtsev *et al.*, 2006), is also shown. (b) Schematic of the FCV p32, p39 and p30 coding regions highlighting the potential hydrophobic regions which may function in membrane association.

Membrane preparations from cells infected with FCV are capable of RNA synthesis *in vitro*, indicating that all the components necessary for FCV RNA genome replication are membrane-associated (Green *et al.*, 2002). Furthermore, these membranes contained the mature non-structural proteins p32, p39 and p30, produced by processing of the viral polyprotein synthesized from open reading frame 1 (Fig. 1a[Fig f1]), implying a role in replication (Green *et al.*, 2002). This also appears to be the case for MNV since all the viral non-structural proteins were shown to co-localize with the site of virus replication (Hyde *et al.*, 2009).

In this paper, we demonstrate that p32, p39 and p30 are integral membrane proteins and, when expressed in cells, localize to the ER. In addition, expression of p39 or p30 results in gross reorganization of the ER as well as altering ER homeostasis.

## RESULTS

### p32, p39 and p30 contain potential membrane-spanning regions

Bioinformatic analysis of the FCV p32, p39 and p30 sequences using the online TMPred server (Hoffman & Stoffel, 1993) identified a number of hydrophobic regions predicted to function as membrane-spanning regions (Fig. 1b[Fig f1]). Transmembrane helices were predicted for residues 229–247 of p32, for residues 6–23, 33–58 and 207–227 of p39 and for residues 180–203 and 249–267 of p30. The prediction of each potential transmembrane region was confirmed by at least one independent program (from DAS, TmPro and Memsat3; see Supplementary Table S1, available in JGV Online).

### Enhanced cyan fluorescent protein (ECFP)-tagged p32, p39 and p30 behave as integral membrane proteins

**Fig. 2. f2:**
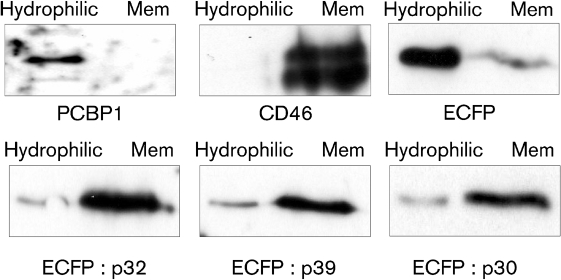
FCV p32, p39 and p30 proteins are capable of targeting a heterologous protein to the hydrophobic fraction. Western blot analysis of the hydrophilic and membrane (Mem) fractions prepared from cells transfected with ECFP and fusions of ECFP with the FCV p32, p39 and p30 proteins.

To determine if the FCV p32, p39 and p30 proteins contained membrane targeting sequences, the viral proteins were fused to the C terminus of ECFP and expressed in cells. Membrane association was determined using the Mem-PER kit (Pierce) which is based on TX-114 partitioning (Qoronfleh *et al.*, 2003) followed by Western blot analysis (Fig. 2). As expected, the nucleic acid binding protein PCBP1, previously reported to be largely cytoplasmic (Berry *et al.*, 2006), was found almost exclusively in the hydrophilic fraction, whereas CD46, a type 1 membrane protein (Liszewski *et al.*, 1991), was found in the membrane fraction (Fig. 2). While >90 % of native ECFP was found in the hydrophilic fraction, the ECFP : p32, ECFP : p39 and ECFP : p30 were largely associated with the hydrophobic membrane-bound fraction (Fig. 2).

**Fig. 3. f3:**
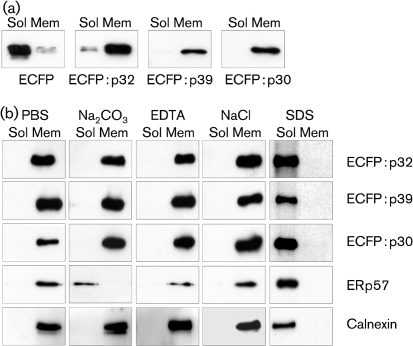
FCV p32, p39 and p30 proteins behave as integral membrane proteins. (a) Western blot analysis of soluble (Sol) or membrane (Mem) fractions from cells transfected with ECFP or ECFP p32, p39 and p30 fusion proteins. (b) Western blot analysis of membrane preparations subsequently extracted with either PBS, Na_2_CO_3_, EDTA, NaCl or SDS to examine the mechanism of association of the FCV p32, p39 and p30 proteins. Membranes from transfected cells were prepared as in (a), extracted with the various reagents and collected by high-speed centrifugation. Membrane-associated and solubilized proteins were then analysed by Western blot analysis using either anti-EGFP, anti-ERp57 or anti-Calnexin antisera.

In a second experiment, post-nuclear membrane preparations were extracted with buffers to discriminate between peripheral membrane and integral membrane association. Native ECFP was again detected in the soluble fraction and the ECFP : FCV fusion proteins were found associated with membranes (Fig. 3a[Fig f3]). Membrane fractions were then treated with NaCl, EDTA or Na_2_CO_3_ to remove peripheral membrane proteins, or extracted with 1 % SDS (Fig. 3b[Fig f3]). Na_2_CO_3_ released the endogenous ER lumen protein ERp57 (Fujiki *et al.*, 1982), but not resident ER integral membrane protein calnexin (Hebert *et al.*, 1995) which could only be released by 1 % SDS. The FCV p32, p39 and p30 ECFP fusion proteins behaved in an identical manner to calnexin, being released from membrane preparations only after treatment with 1 % SDS, indicating that they are integral membrane proteins (Fig. 3b[Fig f3]).

### Untagged FCV p32, p39 and p30 proteins localize to the ER

**Fig. 4. f4:**
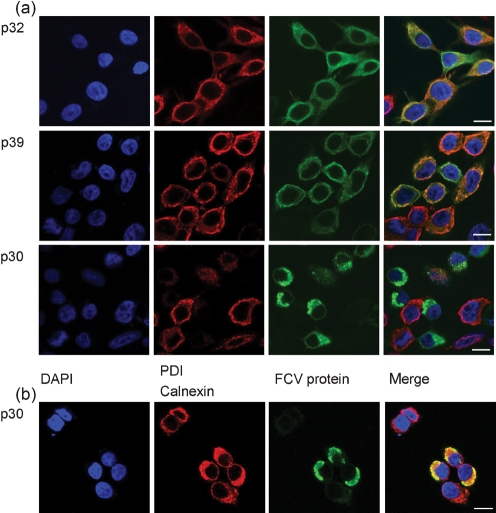
FCV proteins p32, p39 and p30 co-localize with ER markers in transfected cells. (a) FCV proteins p32 and p39 co-localize with the ER marker PDI in transfected cells. The FCV protein p30 resulted in a reduction in the visible PDI signal. (b) FCV p30 protein co-localized with the ER marker calnexin in transfected cells. Cells were fixed and stained as detailed in Methods. Bars, 10 μm.

The subcellular location of untagged FCV proteins expressed alone in 293T cells was examined by confocal laser scanning microscopy (Fig. 4). Although derived from a different organism to the natural host for FCV, it is worth clarifying that these cells have been shown to support FCV infection and release of infectious virus when expressing the feline JAM1 receptor for FCV (Makino *et al.*, 2006). The untagged p32 and p39 proteins co-localized with PDI, suggesting ER-localization for these proteins (Fig. 4a[Fig f4]). FCV p39 also resulted in an apparent condensation of the PDI signal and partial loss of ER structure when compared with normal ER distribution at higher magnification (data not shown). Expression of p30 reduced the signal for PDI, possibly by preventing access of the primary antibody to fixed samples, or causing loss of PDI from the ER. ER localization of FCV p30 was confirmed when antibodies to calnexin were used, and resulted in evident polarization of the ER (Fig. 4b[Fig f4]). Control images of untransfected 293T cells immune-stained for PDI and calnexin are provided for reference in Supplementary Fig. S1(a).

Contextual evidence for the ER association of the FCV p39 and p30 proteins was obtained in virus-infected Crandel–Rees feline kidney (CRFK) cells. Labelling of infected cells with antibodies specific to p39 or p30 with calnexin demonstrated partial co-localization of these proteins (Supplementary Fig. S1b).

### Untagged FCV p30 and p39 perturb accumulation of a transiently expressed ER marker HRP^KDEL^

The marked effects of FCV p39 and p30 expression on host cell ER distribution and PDI signal prompted an investigation into how these proteins affect ER function. Numerous reports have indicated that picornavirus non-structural proteins are capable of interfering with host cell ER function such as FMDV 2C which blocks secretion, and the 2BC precursor which blocks transport from the ER to the Golgi (Moffat *et al.*, 2005, 2007); however, little is known in this respect about the calicivirus non-structural proteins.

**Fig. 5. f5:**
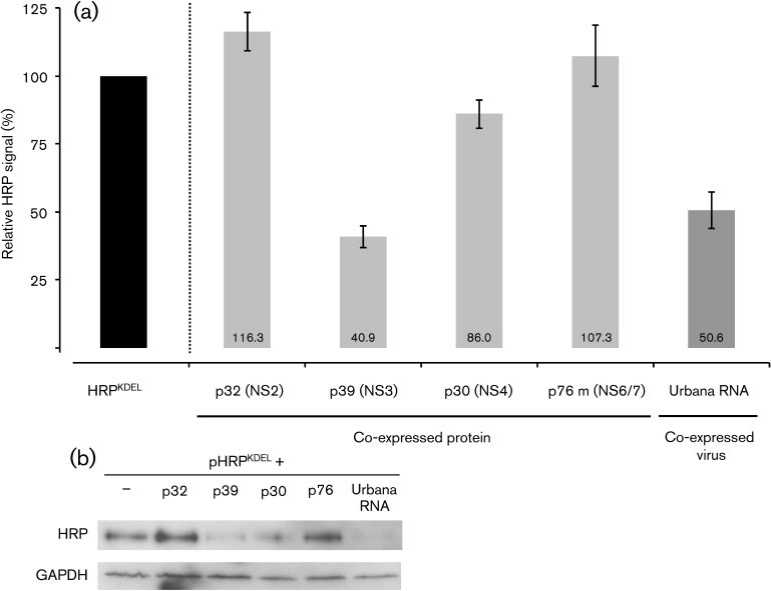
FCV p30 and p39 proteins affect the accumulation of a transiently expressed HRP protein containing the KDEL ER retention signal, as does FCV replication. (a) Cells expressing HRP^KDEL^ were colorimetrically assayed 24 h post-transfection using ELISA substrate. Absorbance was measured at 450 nm. The average signal observed during co-expression of the FCV p32, p39, p30 and p76 m proteins as well as during FCV replication following viral RNA transfection is presented as a percentage of the HRP^KDEL^/blank plasmid control. Error bars indicate sem from experiments and colorimetric assays performed in triplicate. (b) Western blot of the same lysates performed using anti-myc antibody to detect the HRP^KDEL^ and anti-GAPDH.

The effects of FCV proteins on the integrity of the ER were examined by following the location of horseradish peroxidase (HRP) carrying a C-terminal KDEL ER retention signal. The KDEL signal on ER-resident proteins is recognized by KDEL receptors in the *cis*-stack of the Golgi, resulting in retrograde transport back to the ER (Lewis & Pelham, 1992). The effects of FCV proteins on the levels of cell-associated HRP^KDEL^ are shown in Fig. 5. Co-expression of FCV p39 with HRP^KDEL^ significantly reduced the HRP activity in cells (by 59 %; Fig. 5). The FCV p32 and p30 protein showed no significant (±20 %) effect on HRP activity (Fig. 5). The effect of another FCV protein p76 m, a protease polymerase fusion lacking the self-catalytic cleavage sequence but maintaining polymerase function (Wei *et al.*, 2001), was also assayed as a negative control; this protein showed no effect on HRP accumulation when compared with cells singularly transfected with HRP^KDEL^ (Fig. 5a[Fig f5]). Western blot detection of the HRP^KDEL^ protein in the same experimental lysates demonstrated a correlation between total HRP and the active HRP quantified by colorimetric assay (Fig. 5b[Fig f5]). Interestingly, however, any reduction in HRP enzymic activity was associated with a marked reduction in observable HRP by Western blot. Analysis of the supernatants from all transfections for secreted HRP failed to detect any difference over control (data not shown). Western blot analysis showed equal levels of GAPDH levels indicating that co-expression of p32, p30 and p39 with HRP^KDEL^ did not markedly increase cell death or have a gross effect on host cell gene expression (Fig. 5b[Fig f5]). These observations were confirmed by confocal microscopy using a fluorescent substrate of HRP (data not shown). Of note, the previously described distribution of FCV p30, p32 and p39 was again observed using immunofluorescence, indicating that expression of HRP^KDEL^ had no effect on FCV p32, p39 and p30 localization.

### FCV replication reduces HRP^KDEL^ levels

The experiments above were repeated but cells were transfected with a VPg-linked viral RNA to initiate viral genome replication. Cell-associated HRP levels were reduced by 50 % in cells transfected with viral RNA (Fig. 5a[Fig f5], right-hand lane). This reduction was confirmed by Western blot detection of HRP^KDEL^; again, this was markedly reduced (Fig. 5b[Fig f5]) and did not result from significant cell death or inhibition of translation since levels of GAPDH were unaffected by transfection of viral RNA.

### Expression of untagged FCV p30 and p39 in cells results in gross morphological changes to the ER

**Fig. 6. f6:**
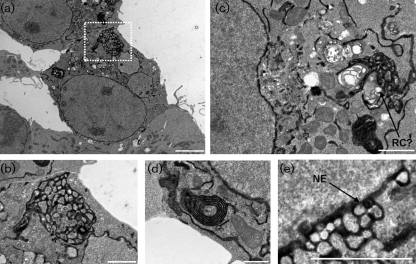
ER and nuclear envelope reorganization observed in FCV p30-expressing 293T cells. Transmission electron microscopy (TEM) of 293T cells expressing the FCV p30 and HRP^KDEL^ proteins obtained 24 h post-transfection. (a) 293T cells at low magnification showing large-scale reorganization of the ER. (b) High magnification image of the boxed area in (a), showing the fenestrated nature of the ER reorganization. (c) 293T cells with extensive ER reorganization, vesicle dilation and putative replication-like vesicle formation. (d) High magnification image of the membrane stacks found frequently in 293T cells transfected with FCV p30. (e) Budding and reorganization of the nuclear envelope in cells transfected with FCV p30. In all panels, the ER-retained HRP^KDEL^ is black due to electron density having being labelled with a heavy metal substrate. NE, Nuclear envelope; RC?, putative replication complex-like structure. Bars, 5 μm (a), 2 μm (b, c, e), 1 μm (d).

**Fig. 7. f7:**
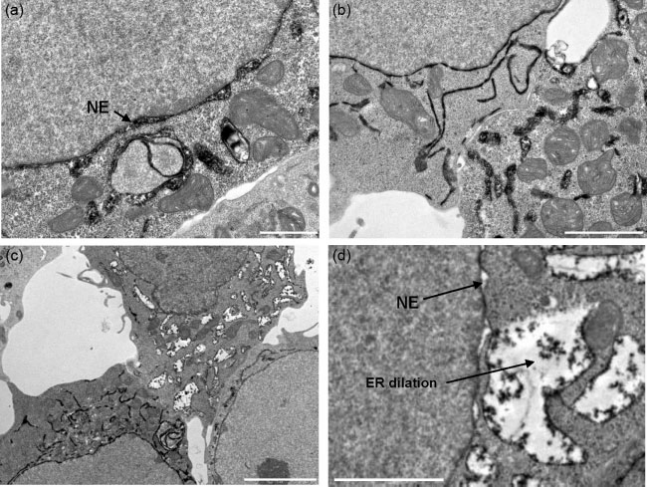
Extensive dilation of the ER in 293T cells expressing FCV p39. TEM of 293T cells expressing the FCV p39 and HRP^KDEL^ proteins obtained 24 h post-transfection. (a) and (b) 293T cells showing moderate dilation of the ER and nuclear envelope following expression of the FCV p39 protein. (c) and (d) 293T cells exhibiting extensive dilation of the ER following expression of the FCV p39 protein. In all panels, the ER-retained HRP^KDEL^ is black due to electron density having being labelled with a heavy metal substrate. NE, Nuclear envelope. Bars, 1 μm (a), 2 μm (b, d), 5 μm (c).

The effects of FCV protein expression on ER ultrastructure were examined by electron microscopy (EM) with HRP activity being monitored using heavy metal substrates (Connolly *et al.*, 1994). 293T cells transfected with HRP alone were used for subsequent comparisons (Supplementary Fig. S2). For all the EM studies, efficient co-expression of both the viral protein and HRP^KDEL^ was confirmed by performing concurrent and equivalent transfections which were subsequently examined by fluorescent microscopy. Co-expression of p32 had no effect on the normal ER distribution seen in singularly transfected HRP^KDEL^ cells (Supplementary Fig. S3). p30 expression appeared to cause significant reorganization of the ER into large, concentrated, fenestrated networks (Fig. 6a–c[Fig f6]); these are usually observed as being polarized to one side of the cell. In addition, a significant amount of multi-layered membrane stacks were observed (Fig. 6d[Fig f6]) as well as potential replication vesicles (Fig. 6c[Fig f6]). In some cases, dilation/budding of the nuclear envelope was also observed (Fig. 6e[Fig f6]). In contrast, expression of p39 caused extensive dilation of the ER and nuclear envelope associated with a reduction in the HRP signal (Fig. 7). Cells were observed with varying degrees of ER and nuclear envelope dilation – from small disruption of the regular condensed HRP signal (Fig. 7a, b[Fig f7]) to significant loss of HRP signal – associated with large, distinct and dilated ER bodies (Fig. 7c, d[Fig f7]).

### Infected CRFK cells exhibit similar ER reorganization

The HRP^KDEL^ marker was used to observe the effects of FCV replication on the structure of the ER in feline CRFK cells. Uninfected CRFK cells expressing HRP^KDEL^ alone were used for subsequent comparisons (Supplementary Fig. S2). To ensure all cells expressing HRP^KDEL^ were efficiently infected, an m.o.i. of 5 was used. Analysis of HRP^KDEL^-positive cells demonstrated the characteristic and previously recorded signs of infection were all evident, such as rounding of the cells, accumulation of numerous smooth replication vesicles in the cytoplasm, dilation of the ER and nuclear envelope as well as paracrystalline arrays of virus (approx. 35 nm in diameter) (Supplementary Fig. S4) (Green *et al.*, 2002; Studdert & O'Shea, 1975).

**Fig. 8. f8:**
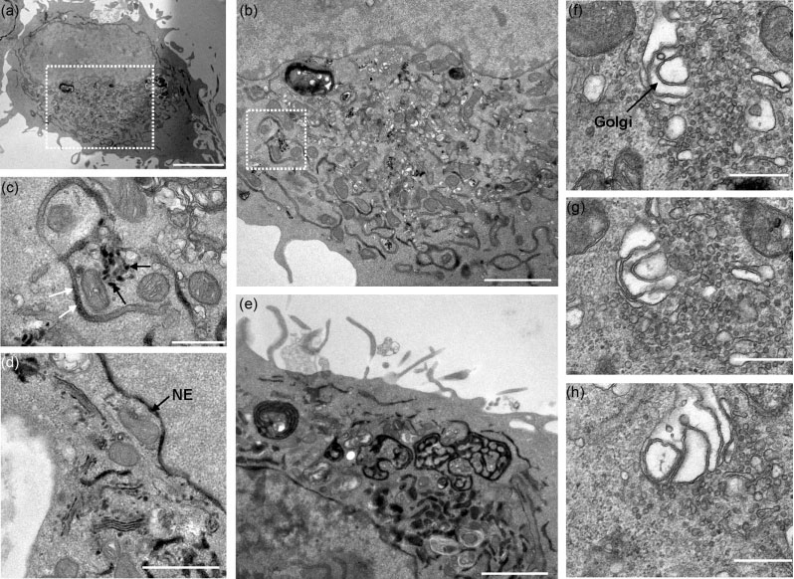
FCV-infected CRFK cells expressing the ER-retained HRP^KDEL^ protein (black) exhibit characteristic signs of calicivirus infection and show correlation to transient protein studies in 293T cells. TEM of CRFK cells expressing the HRP^KDEL^ protein and infected with FCV (Urbana) at an m.o.i. of 5. (a) Low magnification image of FCV-infected cells showing enlarged and circularized morphology, organelle polarization, vesicle formation and reduced HRP^KDEL^ signal. (b) Enlarged image of the boxed area in (a). (c) Enlarged image of the boxed area in (b) showing ER dilation (white arrows), an associated loss of ER resident HRP^KDEL^ signal and putative budding/release of HRP^KDEL^ from the ER (black arrows). (d) High magnification image of FCV-infected CRFK cells showing accumulation of HRP^KDEL^ in all stacks of the Golgi apparatus. (e) Infected CRFK cells exhibiting membranous ER structures analogous to those seen in 293T cells expressing FCV p30. (f–h) A proportion of infected CRFK cells exhibited dilation in stacks of the Golgi apparatus. Images are representative images taken from a Z stack series through the cell. NE, Nuclear envelope. Bars, 5 μm (a), 2 μm (b, e), 500 nm (c, f, g, h), 1 μm (d).

Polarized fenestrated ER observed after expression of p30 in 293T cells were also clearly evident (Fig. 8e[Fig f8]) as were the abundant membrane stacks (Fig. 8e[Fig f8]). The extensive dilation of the ER caused by p39 was not evident although in some instances, partial dilation and loss of HRP signal was observed in both the ER (Fig. 8c[Fig f8]) and nuclear envelope (data not shown). Significantly, our observations suggest that as infection develops, the HRP^KDEL^ signal is lost (Figs 5 and 8a[Fig f8]) making identification of potential HRP-labelled replication complexes problematic. However, HRP was detected in small 40–60 nm vesicles, potentially transitional elements, adjacent to unlabelled ER (Fig. 8c[Fig f8]).

Of interest, in cells displaying morphological signs of infection, we also observed abnormal effects on the Golgi apparatus. HRP was detected throughout all the folded stacks of the Golgi (Fig. 8d[Fig f8]), whereas in uninfected cells, expression could only be found in the first *cis*-stack (Supplementary Fig. S2). In other cells, the Golgi was severely disrupted with individual stacks being extensively dilated (Fig. 8f–h[Fig f8], for reference data showing undisturbed Golgi see Supplementary Fig. S2).

## DISCUSSION

Small RNA viruses, such as picornaviruses and caliciviruses, are known to form their replication complexes on proliferated membranous vesicles in the cytoplasm (Belov & Ehrenfeld, 2007; Belov *et al.*, 2007; Green *et al.*, 2002; Schaad *et al.*, 1997). These membrane-associated replication complexes have been isolated from FCV-infected cells and shown to be capable of viral RNA synthesis and to contain an abundance of both structural and non-structural viral proteins (Green *et al.*, 2002). The presence of p32, p30 and p39 in these replication complexes, together with our previous data showing interactions between these proteins, highlights an integral role for these proteins in replication complex formation and function (Green *et al.*, 2002; Kaiser *et al.*, 2006).

Work on different families of picornaviruses agree that the 2B, 2C and 3A proteins are critical for the formation of virus-induced vesicles (Aldabe & Carrasco, 1995; Bienz *et al.*, 1983, 1992; Cho *et al.*, 1994; Taylor & Kirkegaard, 2008). Our findings show that FCV p32, p39 and p30 bind membranes and induce membrane rearrangements, making them potential orthologues of the picornavirus 2B, 2C and 3A proteins. FCV p30 may be a functional orthologue of poliovirus/enterovirus 3A since both proteins cause swelling of the ER and colocalize with calnexin and, similarly to FMDV 3A, polarize to one side of the cell (Doedens *et al.*, 1997; Garcia-Briones *et al.*, 2006; Taylor & Kirkegaard, 2008). The amino acid sequences and properties of 3A proteins vary considerably between picornaviruses. The 3A proteins of enteroviruses, for example, bind the ER and block ER-to-Golgi transport, but this property is not shared by the 3A proteins of other genera. Functionally, the FCV p39 protein more closely resembles the 2B protein of some other picornaviruses studied to date, although based on sequence alone, it is the 2C orthologue (36 % amino acid conservation when compared with the poliovirus 2C protein). FMDV 2B and 2BC locate to the ER when expressed alone in cells and, as seen for p39, cause a swelling of ER cisternae (Moffat *et al.*, 2007).

Functional orthologues of 2C were less easy to define. Unlike the FCV membrane proteins examined in this study, work to date suggests that the 2C proteins of picornaviruses affect the Golgi stacks, rather than the ER. Poliovirus 2BC disrupts the Golgi stacks and generates small clusters of empty vesicles (Cho *et al.*, 1994; Suhy *et al.*, 2000). Although FCV p32, p30 and p39 individually appeared not to disrupt the Golgi, infection with FCV did lead to significant alteration of this organelle (Fig. 8f[Fig f8]). While our studies draw similarities between the effects of picornavirus and FCV replicase proteins on membrane rearrangements in cells, it is important to note that the generation of a functional replication complex requires the coordinated assembly of several replicase proteins on membranes. Previous studies clearly show that different combinations of replicase proteins can induce different kinds of membrane rearrangement. In the case of poliovirus, for instance, expression of both 2BC and 3A is required to generate the double-membraned vesicles seen in infected cells.

FCV infection and expression of p30 and p39 caused a reduction in the signal from luminal ER proteins such as PDI. Similar reductions in PDI signal were observed for both swine vesicular disease virus and FMDV as a result of viral protein expression, specifically 3A (Martin-Acebes *et al.*, 2008). It is possible that assembly of FCV replicase proteins on the ER affects the KDEL recycling pathway, or results in retrograde translocation of luminal ER proteins into the cytosol. Retrograde translocation preferentially targets misfolded proteins for transport into the cytosol for degradation (Raasi & Wolf, 2007). Normally, ER accumulation of HRP^KDEL^ is supported by retrograde transport from the Golgi to the ER following KDEL receptor recognition in the low pH conditions of the *cis*-stack of the Golgi. The KDEL receptor is then thought to associate with COPI and transport back to the ER where the neutral pH promotes dissociation of HRP^KDEL^ from the KDEL receptor (Murshid & Presley, 2004). Abnormal accumulation of the HRP^KDEL^ in multiple stacks of the Golgi in infected cells (Fig. 8d[Fig f8]) and extensive disruption of the Golgi in some cells (Fig. 8f–h[Fig f8]) indicates that this process is somewhat inhibited, although interestingly we did not detect secreted HRP (data not shown). Studies on poliovirus and coxsackievirus show that the enterovirus 3A proteins are able to modulate the function of the Arf GTP exchange factors BigG1/2 and GBF1 that allow the Arf1 GTPase to recruit *β*-COP to form the COPI coats required for retrograde HRP^KDEL^ transport. Inhibition of Arf-1 function results in disassembly of the Golgi (Beske *et al.*, 2007) and similar Golgi disassembly has been reported following examination of FCV-infected cells by EM (this study and Green *et al.*, 2002). It will be interesting to determine whether FCV and/or p39 similarly affect the activity of Arf 1 and COPI coat assembly, and in this way prevent the return of luminal ER protein to the ER, and/or lead to the Golgi fragmentation we have observed in infected cells.

Arf-GTPases are implicated in the development of viral replication vesicles since some small RNA viruses (such as poliovirus) are BFA sensitive, a known inhibitor of these proteins (Belov & Ehrenfeld, 2007; Belov *et al.*, 2007; Maynell *et al.*, 1992). Arf–GEFS promote formation of Arf–GTP which is required to generate COPI-coated vesicles needed for retrograde transport from the Golgi to the ER. Replication of FCV is not sensitive to BFA, implying that replication does not require Arf–GTP and/or COPI-coated vesicles (Green *et al.*, 2002). Similarly, MNV has been shown to be insensitive to BFA treatment (Hyde *et al.*, 2009). These authors suggest that MNV may adopt a BFA-independent mechanism of blocking ER–Golgi transport and utilizing host cell membranes to establish replication vesicles (Hyde *et al.*, 2009). This is supported by MNV co-localization with elements of the ER (calnexin), *cis*-/medial-Golgi (giantin), *trans*-Golgi (GalT) and endosomes (EEA1). Our research points to an ER-derived origin for the membranous vesicles since FCV p32, p39 and p30 all localize to this organelle when expressed individually; however, the observed disassembly of the Golgi in infected cells cannot exclude an accessory role for this organelle and our data would lead us to suggest that the initiation step of replication complex formation takes place on the ER but various other components of the secretory pathway may become involved as the replication complex matures.

Several recent studies have linked picornavirus replication to the induction of autophagy and autophagosomes that deliver cytosolic content to lysosomes for degradation. It is unclear whether autophagy is activated as a bystander defence against infection leading to degradation of replicase proteins, or actively induced by viruses to provide a niche for replication (Wileman, 2006). Autophagy is activated during poliovirus and coxsackievirus infection and this increases virus yields (Taylor & Kirkegaard, 2008; Wong *et al.*, 2008). Results suggest that autophagosomes may provide a platform for replication or perhaps in some way facilitate cellular exit of viruses without lysis (Jackson *et al.*, 2005; Taylor & Kirkegaard, 2008). The role played by autophagy in FCV replication remains to be investigated in detail, but EM studies show that, in common with FMDV-infected cells (Monaghan *et al.*, 2004), FCV generates significantly less double-membraned vesicles (our observations and Green *et al.*, 2002) when compared with poliovirus.

One potential consequence of FCV protein-induced membranous reorganization is a loss of ER homeostasis, induction of the unfolded protein response (UPR) and apoptosis, the latter has been recorded for FCV (Natoni *et al.*, 2006; Roberts *et al.*, 2003; Sosnovtsev *et al.*, 2003). Japanese encephalitis virus has been shown to trigger the UPR to alleviate virus-mediated sensitivity or induce UPR-related chaperone activation and membrane proliferation (Su *et al.*, 2002; Yu *et al.*, 2006). However, preliminary data would indicate that splicing of the Xbp1 mRNA, one marker for UPR activation, is not altered during FCV infection (data not shown). Whether this reflects no specific involvement for the UPR in infection or, in contrast, viral inhibition of UPR activation in response to the ER stress potentially caused by p30 or p39 is unclear at this juncture, although this represents an interesting point for further research.

The specific aspects of the complex cellular trafficking pathways that FCV undermines to mediate replication complex formation are still not entirely clear but our data would clearly indicate that three of the non-structural proteins can target the ER and of these, two result in various effects on ER structure/function. Ongoing studies focus on the characterization of the mechanism of calicivirus replication complex formation and the cellular targets modified by the membrane-bound calicivirus non-structural proteins. This work gives further insights into how this family of important pathogens subvert the host cell to lead to viral replication.

## METHODS

### Viruses and cells.

Human embryo kidney 293T cells and CRFK cells were maintained at 37 °C/10 % CO_2_ using Dulbecco's modified Eagle's medium containing 10 % fetal calf serum, penicillin (100 SI units ml^−1^) and streptomycin (100 μg ml^−1^). FCV Urbana strain was cultivated and titrated in CRFK cells.

### Membrane protein fractionation.

The membrane fraction from 5×10^7^ cells transfected with the relevant expression constructs was isolated using the Mem-PER membrane protein extraction kit (Pierce) following the manufacturer's instructions. This procedure is based on differential membrane protein solubility in Triton X-114, a non-ionic detergent, as described previously (Bordier, 1981).

### Membrane association.

293T cells were transfected with plasmids encoding ECFP or fusions of ECFP to the FCV p32, p39 and p30 proteins [amplified from the FCV Urbana strain full-length clone pQ14 described by Sosnovtsev *et al.* (2002) – primer details available upon request]. Forty hours after transfection, membranes were prepared and the association of the ECFP fusions with membranes was examined using a well-established protocol which discriminated against peripheral and integral membrane proteins (Fujiki *et al.*, 1982). Briefly, cells were washed twice with ice-cold TES [20 mM Tris (pH 7.4), 1 mM EDTA, 100 mM NaCl]. Samples were kept on ice during the entire procedure. Cells were harvested in 0.5 ml ice-cold TES diluted 1 : 10, collected by centrifugation for 10 min at 4500 ***g***, and resuspended in 250 μl TES diluted 1 : 10. Cells were incubated on ice for 15 min and lysed by 30 strokes in a Dounce homogenizer. Nuclei and cell debris were removed by centrifugation for 10 min at 4500 ***g***. Membrane and cytoplasmic fractions were then generated by centrifugation for 1 h at 150 000 ***g*** at 4 °C. Supernatants were removed and stored at −80 °C until further analysis. Pelleted fractions, containing the membrane-bound proteins were either resuspended in one supernatant volume of lysis buffer [50 mM Tris (pH 7.4), 150 mM NaCl, 1 mM EDTA, 0.1 M phenylmethylsulfonyl fluoride, 1 % Nonidet P-40, 0.05 % SDS] and stored at −80 °C until further analysis or resuspended in 200 μl PBS, 0.1 M Na_2_CO_3_ (pH 11.5), 0.5 M EDTA, 1 M NaCl or 1 % SDS. Resuspended pellet fractions were incubated on ice for 1 h and centrifuged for 1 h at 150 000 ***g*** at 4 °C. The supernatant fractions and the pellet fractions, which were resuspended in 200 μl lysis buffer, were analysed by Western blot.

### Confocal microscopy.

293T cells were transfected using Lipofectamine 2000 (Invitrogen) with 1 μg pTriEx1.1 plasmids encoding either the FCV p32, p39 or p30 proteins. Twenty-four hours after transfection, the cells were fixed with 4 % PFA : PBS, permeabilized using 0.2 % Triton X-100 and blocked using a 1 % BSA : PBS solution. Polyclonal antibodies [raised in guinea pigs as described by Sosnovtsev *et al.* (2002)] directed to the FCV p32, p39 and p30 proteins were used as primary antibodies for immunofluorescence at a 1 : 100 dilution. The cellular ER markers PDI and calnexin were labelled using the anti-PDI and anti-calnexin antibodies [PDI, clone 1D3; calnexin H-70 (sc-11397) Santa-Cruz]. Specific primary antibody binding was identified using AlexaFluor secondary antibodies (Invitrogen). Expression of FCV p39 and p30 proteins in infected CRFK cells was examined using a similar protocol. HRP^KDEL^ was detected using the cyanine 3 fluorescent substrate at a 1 : 300 dilution (tyramide signal assay fluorescence palette system; Perkin Elmer). Images were captured using a Zeiss 510 Meta laser confocal microscope.

### Electron microscopy.

293T cells were transfected with 1 μg pHRP^KDEL^ (Connolly *et al.*, 1994) and/or 1 μg pTRiEx p30, p32 and p39 in 9.5 cm^2^ dishes using Lipofectamine 2000 (Invitrogen). Twenty-four hours later, cells were fixed with 0.5 % glutaraldehyde. CRFK cells were transfected with 1 μg pHRP^KDEL^ in 9.5 cm^2^ dishes using Lipofectamine 2000 (Invitrogen). Eighteen hours later, the cells were infected with FCV (Urbana strain) at an m.o.i. of 5 (or mock-infected in the case of the controls) and left for 6 h before fixation in glutaraldehyde. Cells were subsequently analysed by EM using the methods detailed in the paper by Hollinshead *et al.* (1999); HRP was visualized as recorded by Connolly *et al.* (1994). Images were acquired using an FEI Tecnai G2 electron microscope using a MegaView III CCD camera (Olympus Soft Imaging Solutions).

### HRP^KDEL^ colorimetric assays.

293T cells were transfected with 1 μg pHRP^KDEL^ either alone or with 1 μg pTRiEx1.1 FCV p30, p32, p39, or p76 m in 24-well dishes using Lipofectamine 2000 (Invitrogen). Those cells transfected with pHRP^KDEL^ alone were also transfected with a blank plasmid control (1 μg pTRiEx1.1). Twenty-four hours post-transfection, the cells were lysed in 50 μl RIPA lysis buffer [50 mM Tris/HCl (pH 8.0), 150 mM NaCl, 1 mM EDTA, 1 % Triton X-100, 0.1 % SDS]. Lysate (20 μl) was then added to 50 μl ELISA reagent (TMB liquid; Europa Bioproducts). The reaction was stopped with 50 μl 0.5 M HCl and the absorbance was read at 450 nm. All transfections and colorimetric assays were performed in triplicate. Western blot detection of HRP^KDEL^ was performed using the monoclonal anti-myc antibody (9E10 Santa Cruz) as the HRP is tagged with the myc signal peptide (Connolly *et al.*, 1994). The cellular protein GAPDH was detected using the monoclonal anti-GAPDH antibody (Ambion). Protein loading levels were normalized against cell number in all assays.

### FCV recovery and titration/HRP^KDEL^ colorimetric assays.

293T cells were transfected with 1 μg FCV Urbana VPg-linked RNA prepared as described by Goodfellow *et al.* (2005) either with a blank plasmid control or with 1 μg pHRP^KDEL^ using Lipofectamine 2000 (Invitrogen). Cells were either frozen at 24 h for virus titration or lysed and assayed for HRP/GAPDH as above. Virus titration was performed in CRFK cells by calculating TCID_50_.

## Supplementary Material

[Supplementary material]
