# Supplementary material for: Feline calicivirus p32, p39 and p30 proteins localize to the endoplasmic reticulum to initiate replication complex formation
Source: J Gen Virol. 2010 Mar;91(Pt 3):739–49. doi: 10.1099/vir.0.016279-0 (PMC2885758; doi:10.1099/vir.0.016279-0)
Supplement: [Supplementary material] [file supp_91_3_739__index.html]

 Feline calicivirus p32, p39 and p30 proteins localize to the endoplasmic reticulum to initiate replication complex formation -- Bailey et al. 91 (3): 739 Data Supplement - Supplementary material -- Journal of General Virology

### Feline calicivirus p32, p39 and p30 proteins localize to the endoplasmic reticulum to initiate replication complex formation, by D. Bailey, W. J. Kaiser, M. Hollinshead, K. Moffat, Y. Chaudhry, T. Wileman, S. V. Sosnovtsev and I. G. Goodfellow

*Journal of General Virology* vol. **91**, part 3, pp. 739 - 749

**Supplementary Fig. S1.** Non-transfected cells exhibit regular distribution of the endoplasmic reticulum markers PDI and calnexin, and expression of feline calicivirus p39 and p30 proteins in the virus-infected CRFK cells.

**Supplementary Fig. S2.** 293T cells and CRFK cells transfected with HRPKDEL and subsequently fixed and processed for electron microscopy.

**Supplementary Fig. S3.** Co-expression of FCV p32 and HRPKDEL causes no observable change to the host cell endoplasmic reticulum.

**Supplementary Fig. S4.** Characteristic ultrasound signs of FCV infection were evident in CRFKs expressing HRPKDEL and infected with the Urbana strain of the virus.

**Supplementary Table S1.** Bioinformatic transmembrane domain search results.

[Single PDF] (4.1 MB)
