# Supplementary material for: Feline calicivirus p32, p39 and p30 proteins localize to the endoplasmic reticulum to initiate replication complex formation
Source: J Gen Virol. 2010 Mar;91(Pt 3):739–49. doi: 10.1099/vir.0.016279-0 (PMC2885758; doi:10.1099/vir.0.016279-0)
Supplement: [Supplementary material] [file supp_91_3_739__1.pdf]

(a)

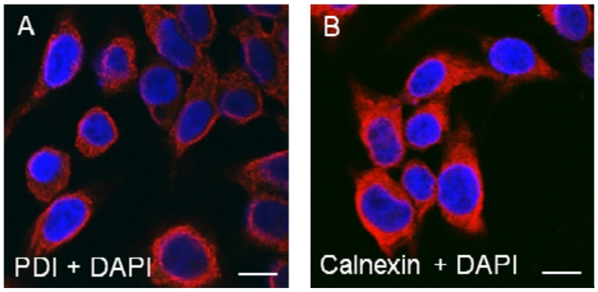

(b)

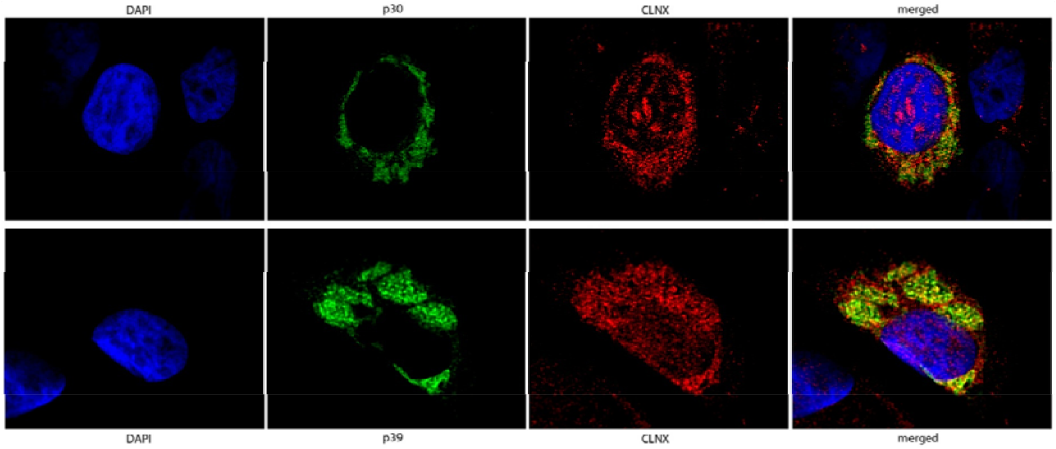

Supplementary Fig. S1. (a) Non-transfected cells exhibit regular distribution of the endoplasmic reticulum (ER) markers PDI and calnexin. 293T cells were treated with Lipofectamine 2000 transfection reagent but not transfected with DNA. Cells were fixed and stained (A, PDI staining; B, calnexin staining) after 24 h as described in Methods. Bars, 10 µm. (b) Expression of feline calicivirus (FCV) p39 and p30 proteins in the virus-infected CRFK cells. Confocal imaging shows co-localization of the p39 and p30 proteins (both green) and ER membrane marker calnexin (red) in the FCV-infected CRFK cells. CRFK cells were infected with FCV at an m.o.i. of 1 and analysed 4 h post-infection by immunofluorescent staining, as described in Methods.

Bailey, D., Kaiser, W. J., Hollinshead, M., Moffat, K., Chaudhry, Y., Wileman, T., Sosnovtsev, S. V. & Goodfellow, I. G. (2010). Feline calicivirus p32, p39 and p30 proteins localize to the endoplasmic reticulum to initiate replication complex formation. *J Gen Virol* **91**, 739–749.

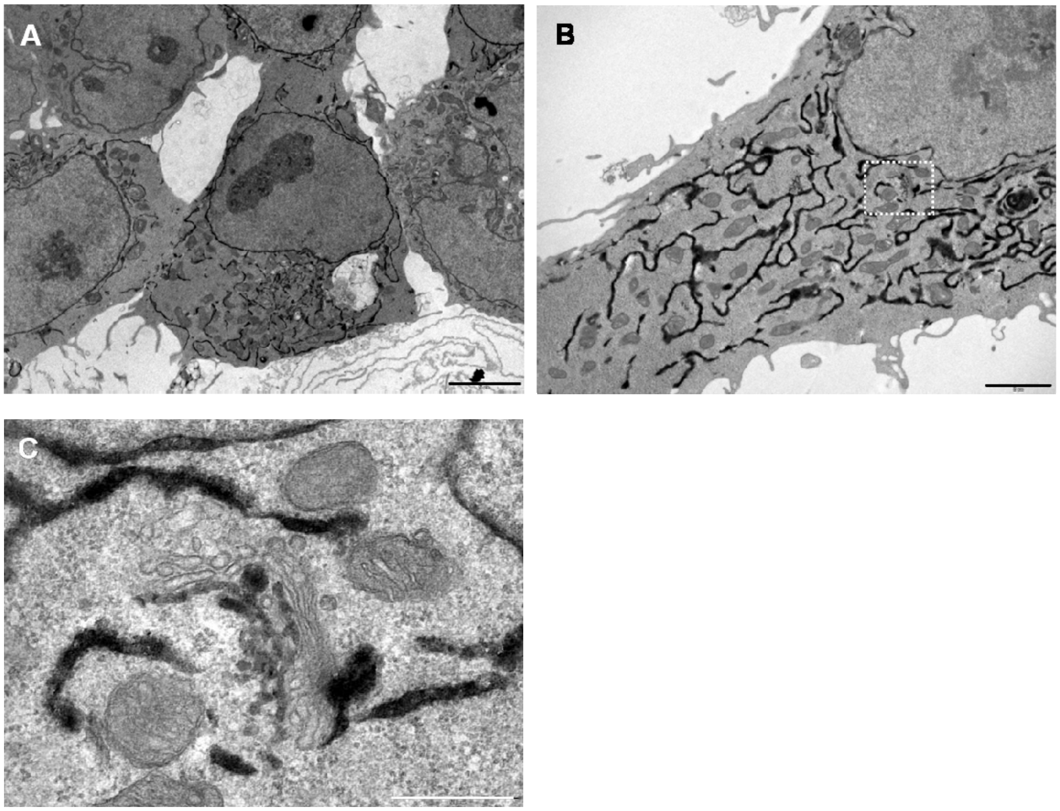

Supplementary Fig. S2. 293T cells (a) and CRFK cells (b) were transfected with HRP^{KDEL} and subsequently fixed and processed for electron microscopy analysis as detailed in Methods. The accumulation of horseradish peroxidase (HRP) in the ER and the organisation of this organelle was typical in all cells examined by EM, as demonstrated by this representative image. (c) Magnified image of boxed area in (b) showing normal Golgi apparatus. Bars, 5 µm (a), 2 µm (b), 500 nm (c).

Bailey, D., Kaiser, W. J., Hollinshead, M., Moffat, K., Chaudhry, Y., Wileman, T., Sosnovtsev, S. V. & Goodfellow, I. G. (2010). Feline calicivirus p32, p39 and p30 proteins localize to the endoplasmic reticulum to initiate replication complex formation. *J Gen Virol* **91**, 739–749.

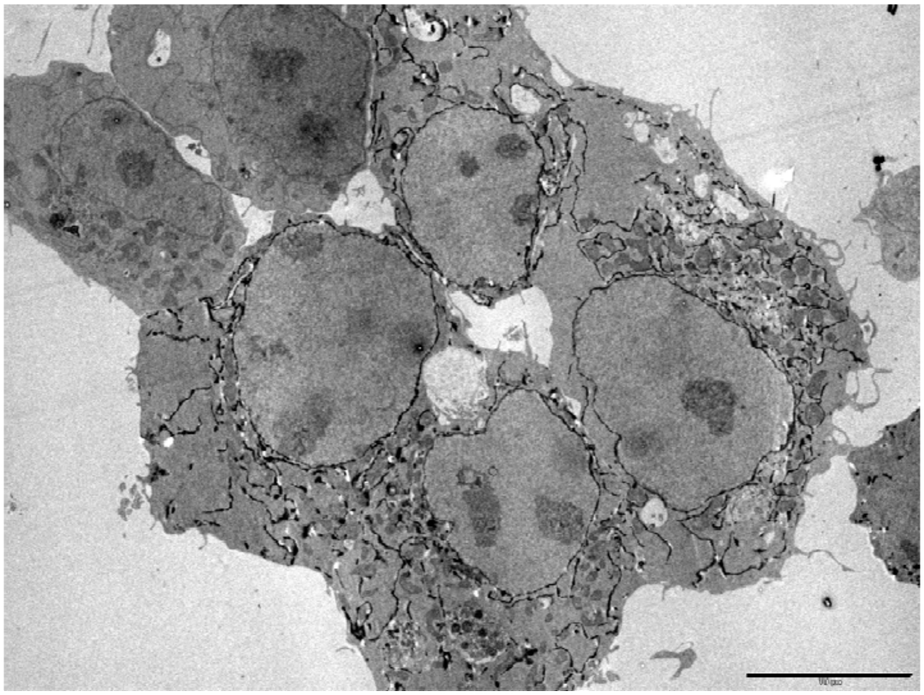

Supplementary Fig. S3. Co-expression of FCV p32 and HRP^{KDEL} causes no observable change to the host cell ER. 293T cells were transfected with HRP^{KDEL} and FCV p32 constructs and subsequently fixed and processed for electron microscopy (EM) analysis as detailed in Methods. The accumulation of HRP in the Er and the organisaton of this organelle was typical in all cells examined by EM, as demonstrated by this representative image. Bar, 10 µm.

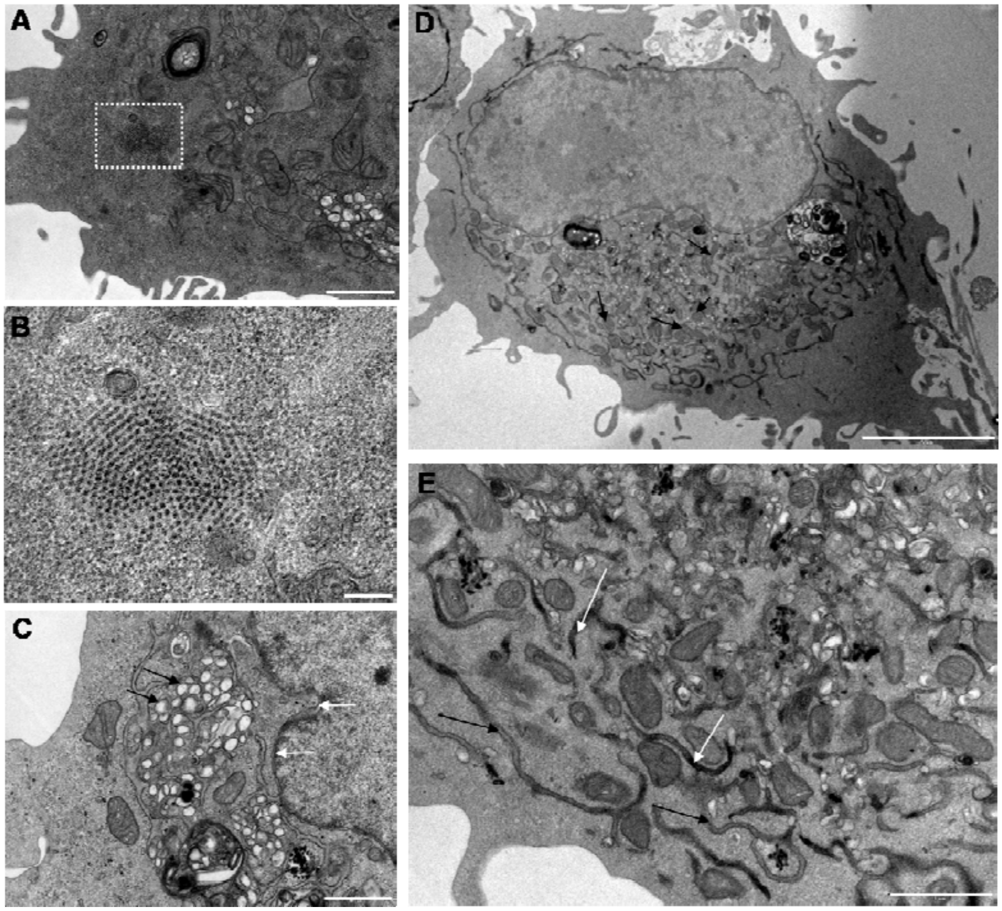

Supplementary Fig. S4. Characteristic ultrasound signs of FCV infection were evident in CRFKs expressing HRP^{KDEL} and infected with the Urbana strain of the virus.(a, b) Low and high magnification images of the cytoplasm of an infected cell shwoing a paracrystalline array of virus particles. The boxed area in (a) is enlarged in (b). (c) High magnification image of the cytoplasm of infected cells showing characteristic replication vesicles (black arrows) and dilation of the nuclear envelope (white arrows). (d) Low magnification image showing rounding of the cell, polarisation of the organelle components and putative mitochondrial recruitment (black arrows). (e) High magnification image of the cytoplasm of an infected CRFK cell showing regions of both positively (white arrows) and negatively (black arrows) HRP-labelled ER. Bars, 1 µm (a, c, e), 200 nm (b), 5 µm (d).

Bailey, D., Kaiser, W. J., Hollinshead, M., Moffat, K., Chaudhry, Y., Wileman, T., Sosnovtsev, S. V. & Goodfellow, I. G. (2010). Feline calicivirus p32, p39 and p30 proteins localize to the endoplasmic reticulum to initiate replication complex formation. *J Gen Virol* **91**, 739–749.

Supplementary Table S1. Bioinformatic transmembrane domain search results

o–i, Outside to inside direction predicted; i–o, inside to outside direction predicted.

Protein	TmPred predicted domain	DAS	TmPro	Memsat 3
p32	aa 229–247 o–i	Yes	No	Yes
p39	aa 6–23 o–i	Yes	Yes	Yes
	aa 33–58 i–o	No	No	Yes
	aa 207–227 o–i	Yes	No	No
p30	aa 180–203 o–i	Yes	Yes	No
	aa 249–267 i–o	Yes	No	Yes
